# Is the heart involved or not? The diagnostic value of cardiovascular magnetic resonance imaging in the assessment of non-cardiac malignancies and metastatic cardiac tumours

**DOI:** 10.1259/bjrcr.20210070

**Published:** 2021-10-05

**Authors:** Catriona Stoddart, Chrysovalantou Nikolaidou, Rachel Benamore, Cheng Xie

**Affiliations:** 1 Department of Radiology, John Radcliffe Hospital, Oxford University Hospitals NHS Foundation Trust and University of Oxford, Oxford, UK; 2 Oxford Centre for Clinical Magnetic Resonance Research, Radcliffe Department of Medicine, John Radcliffe Hospital Oxford, University of Oxford, Oxford, UK

## Abstract

Cardiovascular magnetic resonance (CMR) is a radiation-free, high-spatial resolution technique which provides dynamic assessment of the heart and pericardial tissue. This is particularly useful for the evaluation and characterisation of non-cardiac tumours close to the pericardium for the exclusion of cardiovascular infiltration, and also for the assessment of the extent of myocardial invasion of cardiac metastases. This information can help make key decisions on further management in oncology multidisciplinary meetings. The CMR evaluation and main types of sequences acquired are detailed in this case series to illustrate the application of CMR in the assessment of non-cardiac malignancies and its importance in guiding management.

## Introduction

Cardiovascular magnetic resonance imaging (CMR) is a radiation-free imaging modality able to provide anatomical detail, accurate assessment of myocardial volumes and function, tissue characterisation and dynamic assessment of the heart and pericardial tissue. CMR offers imaging of the heart in any desired imaging plane.^1–5^ These features make CMR an invaluable diagnostic tool in the assessment of non-cardiac tumours within the mediastinum close to the pericardium and metastatic tumours with suspicion of pericardial or myocardial involvement. A particular benefit of CMR is in the determination of cardiovascular infiltration, which can be difficult to assess on other imaging modalities. The results of the CMR scan often provide the decisive information in oncology multidisciplinary meetings (MDMs) for subsequent management. In addition, with the appropriate sequences and selection of the desired imaging planes, it can provide excellent anatomical detail to help with surgical planning for tumours close to but no invading the heart. Being radiation-free CMR, is frequently adopted as the preferred imaging modality for follow-up, especially in young adults.

Some limitations of CMR imaging include the long acquisition time when compared to CT, end-stage renal failure and claustrophobia. However, in these situations, sometimes a time-saving single sequence, or a non-contrast mapping sequences such the T1 and T2-mapping, is adequate to answer the clinical question.^6,7^ These will be discussed in detail in this case series. Absolute contraindications such as metallic foreign bodies, or non-MR compatible pacemakers are important safety checks prior to any MRI scan.

In this case series, we review clinical scenarios in which CMR was used in the evaluation of non-cardiac malignancies to determine cardiovascular involvement. The main objectives are to illustrate CMR protocols, interpretation of the findings, and the role of CMR as an important adjunct to other imaging modalities in clarifying the diagnosis.

## Imaging protocol and technique

CMR can offer invaluable information for the diagnosis and treatment planning of extra cardiac masses or cardiac metastases. More specifically, CMR can provide detailed tissue characterisation, evaluation of vascularity and perfusion of a mass, and assessment for the presence and extent of tissue invasion. Masses should be identified in at least two orthogonal planes and delineated further with different sequences that demonstrate tissue characteristics, using the imaging planes that best visualise the lesion.

A complete CMR protocol includes anatomical imaging of the entire thorax, cine imaging usually using steady-state free precession (SSFP sequences), *T*_1_ weighted and *T*_2_ weighted imaging with fat suppression, early and late gadolinium enhancement imaging. These are useful for localisation, assessment of morphology and margins of the mass, tissue characterisation and evaluation of tumour extension and vascularity. Cine imaging also provides assessment of ventricular function and possible evidence of compression of a cardiac chamber from a mass or due to the presence of pericardial effusion. Early and late gadolinium imaging can identify thrombi inside the heart, as well as scarring or areas of necrosis inside a mass or of the ventricular walls. Although still not widely used and comprehensively evaluated in cardiac or extra cardiac masses, T1- and T2-mapping can add detailed tissue characterisation, without contrast administration.^6,7^ T2 mapping can provide further information about myocardial oedema. Information from parametric mapping techniques can also be displayed as colour maps to facilitate visual interpretation. First-pass perfusion imaging can also be added to the protocol to assess the vascularity of a mass and the risk of bleeding during resection. Finally, CMR tagging sequences can be used in the assessment of the pericardium in cases of unclear mass invasion or infiltration. Of course, the CMR protocol can be adjusted and tailored to the specific mass under investigation.

## Results

### Case 1

**Figure 1.**
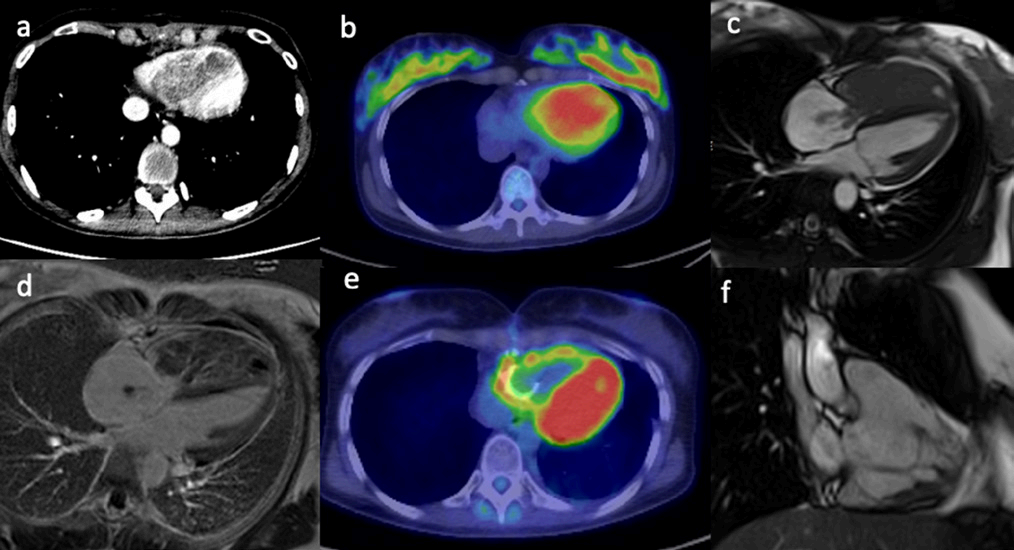
(**a**) Contrast-enhanced CT scan shows an ill-defined low density mass in the right ventricle. (**b**) The PET/CT fused image shows corresponding markedly increased avidity of the right ventricular mass. (**c**) Still frame from SSFP, CMR 4-chamber cine image shows a large mass filling the entire right ventricle with extension of small stalks through the tricuspid valve into the right atrium. (**d**) The late gadolinium enhanced image shows mild heterogeneous enhancement of the mass with some necrotic areas. (**e**) Post-operative PET/CT shows persistent avidity in the right ventricular wall. (**f**) Post-operative, still frame from right ventricular SSFP 3-chamber cine image shows residual irregular mass attached to the inferior right ventricular wall. CMR, cardiac magnetic resonance; PET, positron emission tomography; SSFP, steady-state free precession.

A 36-year-old female with a history of right tibial synovial sarcoma treated with above knee amputation was found to have a large right ventricular mass on routine echocardiography prior to commencing chemotherapy. CT confirmed an ill-defined low-density mass within the right ventricle (Figure 1a) which showed high fludeoxyglucose (FDG) uptake on PET-CT imaging (Figure 1b). CMR was used to further characterise the tumour and showed a large mass filling the entire right ventricle, with parts of the mass protruding into the right atrium through the tricuspid valve in systole (Figure 1c). The mass showed mild heterogeneous gadolinium enhancement with some necrotic areas (Figure 1d). The treatment involved tumour resection and post-operative radiotherapy. Metastasis of biphasic synovial sarcoma was confirmed on histology. A 3 month interval follow-up PET-CT showed increased FDG uptake in the right ventricular wall (Figure 1e), and it was difficult to differentiate between post-operative inflammatory changes or residual tumour. A subsequent CMR identified the presence of residual tumour attached to the inferior right ventricular wall (Figure 1f). Another operation with complete tumour resection was performed.

### Case 2

**Figure 2.**
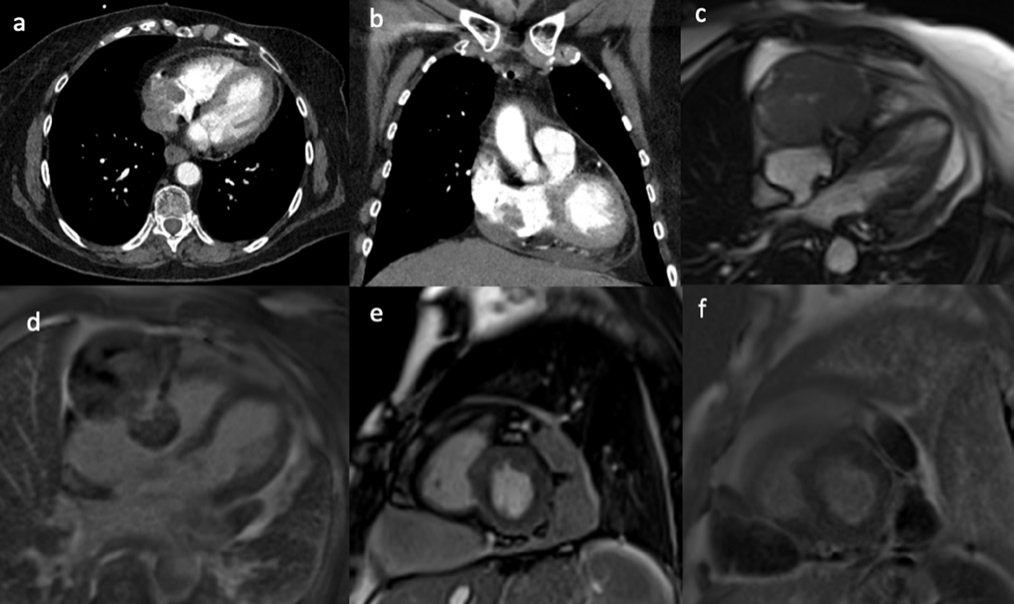
(a, b) CTPA showed an irregular filling defect in the right atrium. The CMR scan acquired 2 months after the CTPA of the same patient. The still frames from SSFP CMR cine 4-chamber (**c**) and the corresponding late gadolinium image (**d**) with the short-axis (**e**) image showing a large ovoid mass adjacent to the right atrium and attached to the right atrioventricular groove, invading into the right atrium. There are also two additional pericardial masses on the left lateral side, not invading into the myocardium, which showed heterogeneous, mild patchy gadolinium uptake on late gadolinium imaging (**f**). CTPA, CT pulmonary angiogram; CMR, cardiac magnetic resonance; SSFP, steady-state free precession.

A 72-year-old female patient presented with gradual onset central chest pain, dyspnoea, and *de novo* atrial fibrillation. Her past medical history included neurofibromatosis Type 1 with multiple resections of related tumours including: left knee myxofibrosarcoma, malignant peripheral nerve sheath tumour of the scalp, gastrointestinal stromal tumour and carcinoma of the left breast. Investigation with computed tomography pulmonary angiogram (CTPA) showed a filling defect in the right atrium (Figure 2a & b). The differential included right atrial thrombus, or the possibility of another tumour related to the patient’s underlying condition. The CMR scan taken 2 months after the CT showed a relatively larger mass adjacent to the right atrioventricular groove mostly situated at the inferior aspect of the pericardium, compressing the right atrium (Figure 2c &d). It also demonstrated two additional masses on the left lateral side of the heart (Figure 2e & f). Unfortunately, the masses were unresectable and the patient was treated with palliative radiotherapy.

### Case 3

**Figure 3.**
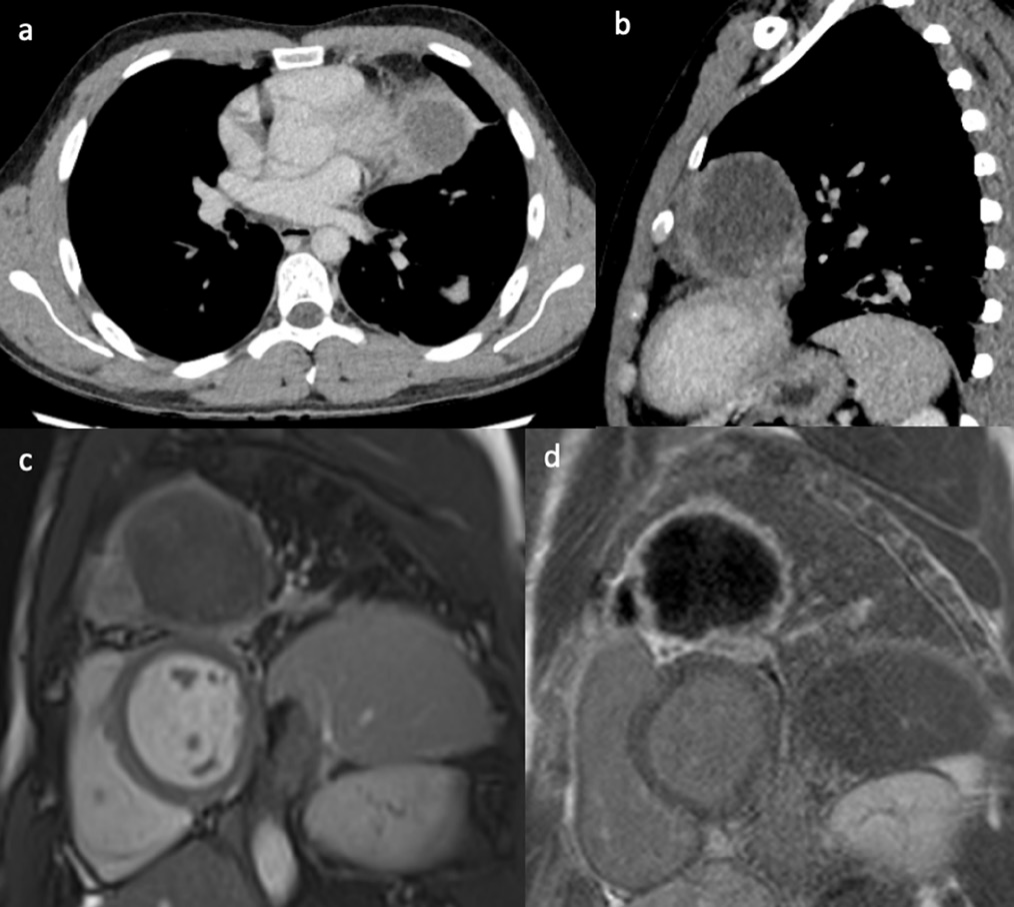
(a, b) Contrast-enhanced CT showing a large left upper lobe lung mass without a clear fat plane between lung and pericardium. Still frame from the short-axis SSFP CMR cine sequence (**c**) and corresponding late gadolinium enhancement image (**d**), showing the lung mass abutting the pericardial surface of the anterior-lateral left ventricular wall. CMR, cardiac magnetic resonance; SSFP, steady-state free precession.

A healthy 27-year-old male patient presented with recurrent haemoptysis. He was found to have a large mass in the left upper lobe on chest CT. The mass was located adjacent to the left ventricular myocardium, and no clear fat plane was visible between the tumour and the pericardial surface (Figure 3a & b), thus raising the suspicion of cardiovascular involvement. The CMR scan (Figure 3c & d) showed that the mass was abutting the pericardial surface without deep invasion. These findings and the images were important for subsequent carthoracic MDM and surgical planning. Subsequent surgery showed that the mass was tethered to the pericardium only and complete resection of the mass was performed. Histology of the tumour showed a mediastinal yolk sac tumour.

### Case 4

**Figure 4.**
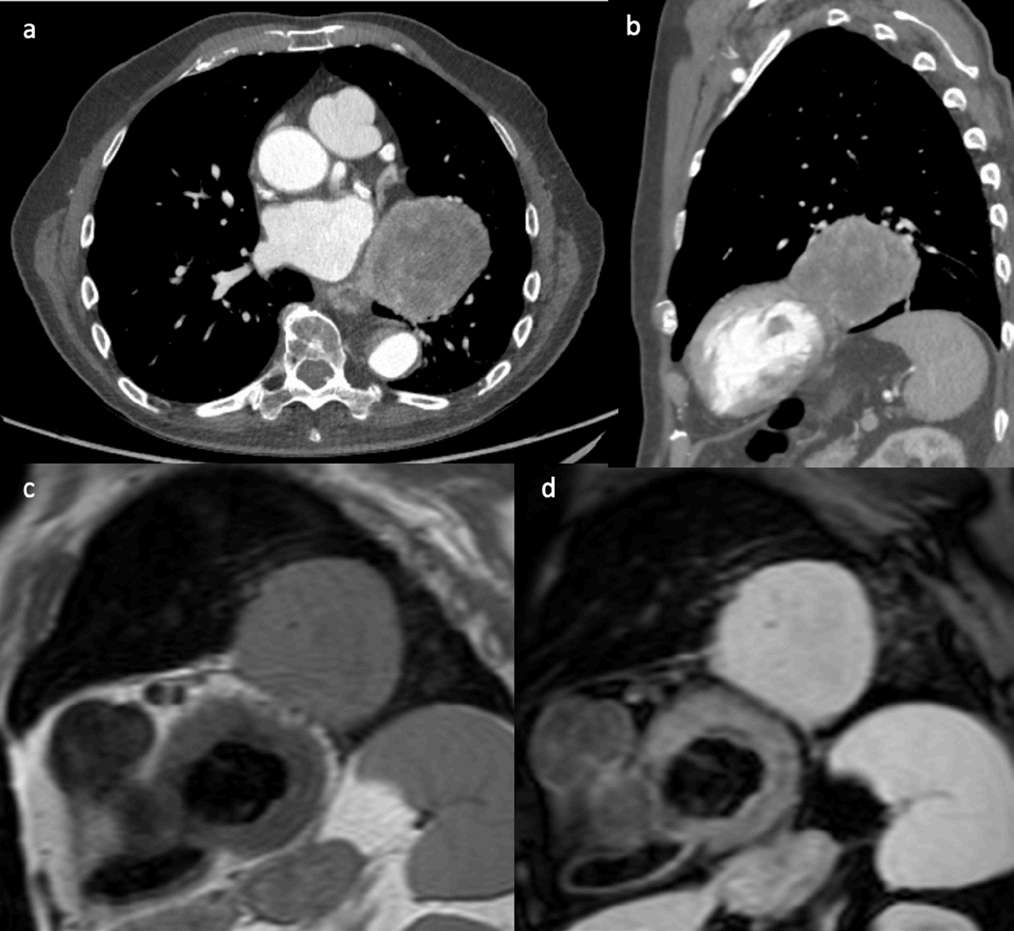
(a, b) Contrast-enhanced CT showed a large retrocardiac left lung mass adjacent to the left ventricular pericardium, raising suspicion of cardiac invasion. *T*_1_ weighted (**c**) and *T*_2_ weighed with fat saturation (**d**) short-axis CMR images showing separation of the mass from the myocardium with a thin pericardial fatty layer. Complete resection of the mass was achieved. CMR, cardiac magnetic resonance.

An 83-year-old male patient presented with persistent dry cough and dysphagia. His CT demonstrated a large retrocardiac left lung mass adjacent to the pericardium of the left side of the heart (Figure 4a & b). As with Case 3, there was a suspicion of cardiovascular involvement given the lack of fat plane between the mass and pericardium. The CMR scan using *T*_1_ weighted and *T*_2_-weighted with fat saturation sequences, demonstrated a thin but clear fat plane separating the tumour from the myocardium (Figure 4c & d). The tumour was completely dissected and released from the pericardium with free margins. Histology confirmed a solitary malignant fibrous tumour.

### Case 5

**Figure 5.**
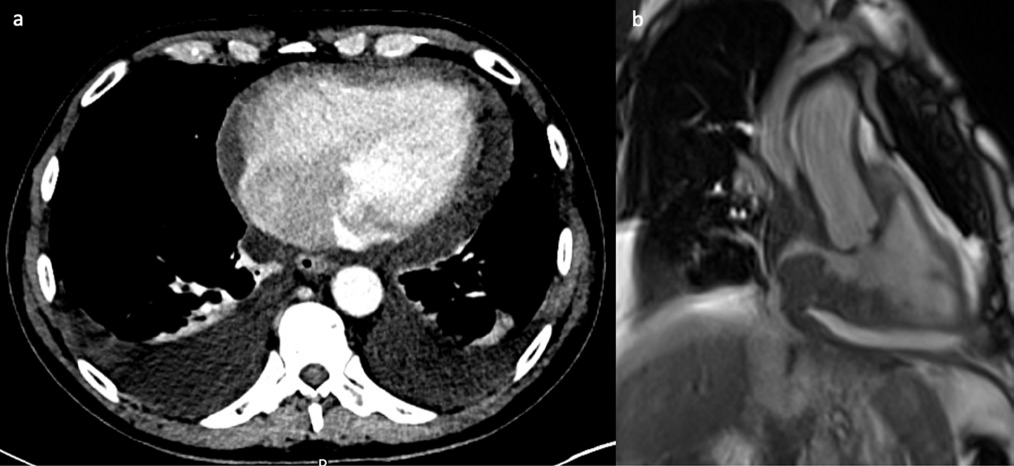
(**a**) Contrast-enhanced CT showing small a pericardial effusion, moderate bilateral pleural effusions and a filling defect in the right atrium. (**b**) Still frame from right ventricular SSFP 3-chamber cine image, showing grossly irregularly thickened atrial walls. The mass extends to involve the venae cavae and causes obstruction to flow of the superior vena cava. SSFP, steady-state free precession.

An 81-year-old male patient with previous B cell lymphoma in remission presented with 1 week history of vomiting, altered bowel habit and general malaise. A body CT was performed as part of the investigation for disease recurrence. The CT showed a small pericardial effusion, moderate bilateral pleural effusions and a subtle filling defect in the right atrium (Figure 5a). The initial working diagnosis was fluid overload with the possibility of a mass in the atrium which required further investigation. An echocardiogram showed bilateral atrial wall thickening with luminal narrowing, which increased the concern of disease recurrence within the right atrium. CMR provided a more definitive diagnosis. There was bi-atrial irregular wall thickening, more pronounced around the right atrial wall. The mass extended into both venae cavae. Cine images clearly demonstrated fixed and also dynamic narrowing of the venous return, especially of the superior vena cava (Figure 5b). Due to the extensive nature of the disease, and fragility of the patient, palliative care was provided.

### Case 6

**Figure 6.**
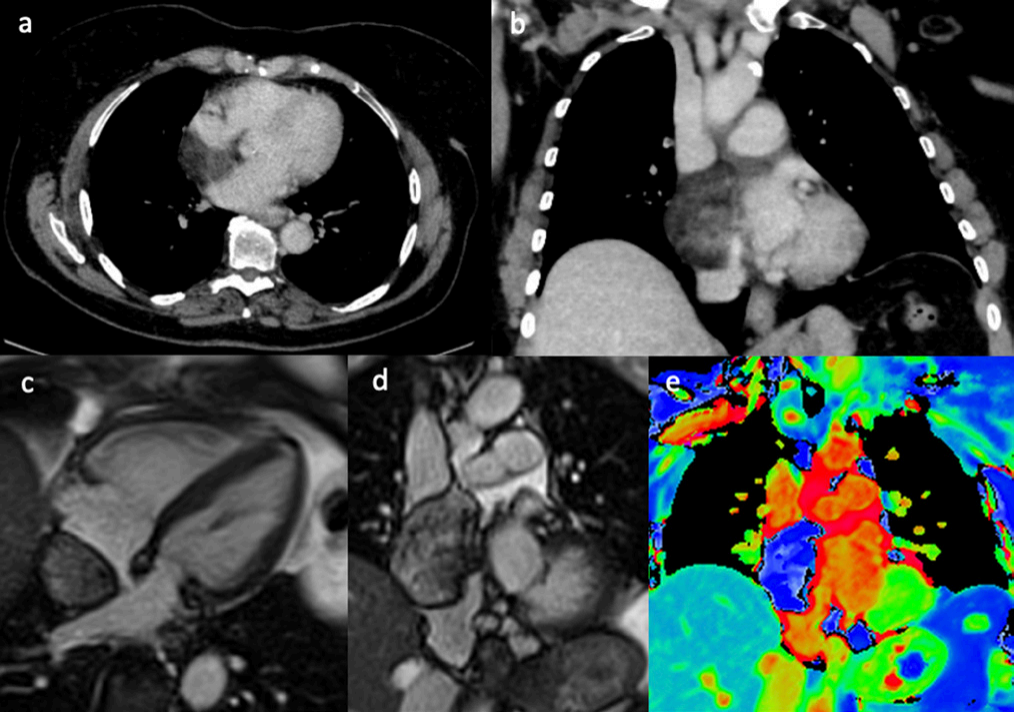
(a & b) Contrast enhanced CT showed an extra atrial but intra pericardial filling defect with fat density. Still frames of SSFP 4-chamber (**c**) and coronal (**d**) cine images, showing an extra cardiac mass abutting the right atrium and located into the venae cavae. The corresponding coronal T1 mapping image (**e**) showing (blue) fat signal within the mass. SSFP, steady-state free precession.

An 81-year-old female patient with previously treated parotid carcinoma underwent routine surveillance CT. The CT showed an extra atrial but intrapericardial filling defect (Figure 6a & b). The area had fat density, which raised the suspicion of a fat containing mass (lipoma, liposarcoma) as parotid carcinoma is considered unlikely to recur in the heart. On CMR, cine image (Figure 6c & d) confirmed the location of the mass as identified on the CT. T1 mapping using the Shortened Modified Look-Locker Inversion recovery (ShMOLLI) sequence was important in characterising the mass composition.^6^ The colour coded images and the low native myocardial T1 values confirmed that the mass had fatty composition, with similar image characteristics (blue colour) as the mesenteric fat, intra- and extrathoracic fat (Figure 6e). As a result, the CMR findings provided further confidence that the mass was a lipoma. Follow-up CMR also showed stable imaging appearance of the mass, and it was treated conservatively as the patient was asymptomatic.

## Discussion

In this case series, we explored the diagnostic value of CMR in the assessment of non-cardiac tumours with suspicion of cardiovascular involvement or cardiac metastases, and demonstrated how CMR can provide the decisive information to guide subsequent management.

In the majority of the cases, depending on the known location of the tumour, the most appropriate cine sequence could provide clear delineation of the mass from the adjacent pericardium and myocardium. In these cases, there is still a discernible MR signal difference between the tumour tissue and the adjacent cardiac tissue and pericardium. In addition, the dynamic nature of the cine images could differentiate the cardiac tissue (which shows systolic/diastolic motion) compared to non-contractile tumour tissue. Other CMR acquisitions such as the *T*_1_ weighted sequences with/without fat saturation, *T*_2_ weighted imaging, and T1 mapping with ShMOLLI were important for detecting fat signals as shown in Case 4 and Case 6, respectively.

This case series used clinical scenarios from the oncology MDMs to illustrate the appropriate use of CMR to answer difficult questions about cardiovascular involvement of extracardiac tumours adjacent to the heart, or the extent of myocardial involvement and resectability of metastatic cardiac tumours. The CMR findings were not only important to guide the decision-making process, but also allowed for surgical planning and follow-up. For Case 3 and Case 4, the cardiothoracic surgeon specifically requested CMR prior to any surgical opinion, which was then followed by evaluation on a case-by-case basis at the MDM. When a patient is referred for CMR for these indications, we would recommend a discussion between the clinical team with the Cardiologist/Radiologist supervising the list to review the clinical background, main clinical objective, and recent imaging to allow careful planning of the scan particularly when only limited sequences can be performed for patients who cannot tolerate the scan or are claustrophobic.

## Learning points

- CMR could provide clear delineation of the mass from the adjacent pericardium and myocardium.
- CMR is an important imaging investigation for the exclusion of cardiovascular involvement from adjacent thoracic tumour.
- Time-saving sequences can be performed for patients who cannot tolerate the scan.
